# Comparative and phylogenetic analysis of chloroplast genomes from ten species in *Quercus* section *Cyclobalanopsis*

**DOI:** 10.3389/fpls.2024.1430191

**Published:** 2024-08-19

**Authors:** Ke Huang, Buyu Li, Xiaoli Chen, Chun Qin, Xuemei Zhang

**Affiliations:** College of Life Sciences, China West Normal University, Nanchong, China

**Keywords:** chloroplast (cp) genome, phylogenetic analysis, *Quercus* section *Cyclobalanopsis*, DNA sequencing, sequence annotation

## Abstract

The genus *Quercus* L. is widely acknowledged as a significant assemblage within East Asia tropical and subtropical broadleaf evergreen forests, possessing considerable economic importance. Nevertheless, the differentiation of *Quercus* species is deemed arduous, and the interrelations among these species remain enigmatic. Leveraging Illumina sequencing, we undertook the sequencing and assembly of the chloroplast (cp) genomes of seven species belonging to *Quercus* section *Cyclobalanopsis* (*Quercus argyrotricha*, *Q. augustinii*, *Q. bambusifolia*, *Q. bella*, *Q. edithiae*, *Q. jenseniana*, and *Q. poilanei*). Furthermore, we collated three previously published cp genome sequences of *Cyclobalanopsis* species (*Q. litseoides*, *Q. obovatifolia*, and *Q. saravanensis*). Our primary objective was to conduct comparative genomics and phylogenetic analyses of the complete cp genomes of ten species from *Quercus* section *Cyclobalanopsis*. This investigation unveiled that *Quercus* species feature a characteristic circular tetrad structure, with genome sizes ranging from 160,707 to 160,999 base pairs. The genomic configuration, GC content, and boundaries of inverted repeats/single copy regions exhibited marked conservation. Notably, four highly variable hotspots were identified in the comparative analysis, namely *trnK-rps16*, *psbC-trnS*, *rbcL-accD*, and *ycf1*. Furthermore, three genes (*atpF*, *rpoC1*, and *ycf2*) displayed signals of positive selection pressure. Phylogenetic scrutiny revealed that the four sections of *Cyclobalanopsis* clustered together as sister taxa. The branch support values ranged from moderate to high, with most nodes garnering 100% support, underscoring the utility of cp genomic data in elucidating the relationships within the genus. Divergence time analysis revealed that Section *Cyclobalanopsis* represents the earliest type of *Quercus* genus. The outcomes of this investigation establish a foundation for forthcoming research endeavors in taxonomy and phylogenetics.

## Introduction

*Quercus* L. is the most diverse genus in *Fagaceae* (Manos et al., 1999), with 430 species worldwide, making it one of the most widely distributed woody genera in the Northern Hemisphere. It has long been closely associated with human ecology (Welter et al., 2012; Gil-Pelegrín et al., 2017). From an ecological conservation and balance perspective, these plants are crucial in maintaining soil structure and fertility, regulating local climatic conditions, and contributing to biodiversity. In terms of economic value and practical applications, the physical properties of these plants, such as high hardness, strength, and good corrosion resistance, provide a solid basis for their use in various industries. Whether the systematic classification of cycads belongs to the genus *Quercus* or is a separate genus within the family Fagaceae has long been debated. The classification of the genus *Quercus* is challenging due to its complex evolutionary history, and it remains an ongoing endeavor (Manos et al., 1999; Kremer et al., 2010; Deng et al., 2018). A novel global phylogenetic framework for the genus *Quercus* has been developed to address this challenge, utilizing nuclear molecular markers and pollen characteristics. This framework divides the genus into two subgenera, *Quercus* and *Cerris*, each further divided into eight sections (Denk et al., 2017). China includes all four previously recognized sections: *Quercus*, *Cerris*, *Cyclobalanopsis*, and *Ilex* (Hubert et al., 2014; Vitelli et al., 2017). Yet, the inclusion of Chinese species in this structure is restricted and significantly less than that of species from North America and Europe.

Historically, the classification of the genus *Quercus* has traditionally been based on morphological features, leaf epidermis characteristics, pollen analysis, and the study of evolutionary processes in a limited group of species (Oh and Manos, 2008; Denk and Grimm, 2009; Deng et al., 2014; Muthreich et al., 2020). Contradictions arose when molecular sequence data were introduced to delineate (sub) sections and series, leading to discrepancies with the classifications previously established through morphological analysis (Denk et al., 2017). For example, investigations using ITS sequences have revealed a close genetic relationship between species grouped under the compound trichome base (CTB) within the section *Cyclobalanopsis* and those in the section *Cerris*, which contradicts conventional morphological taxonomy (Deng et al., 2014). This divergence highlights the lack of a unified approach to classifying the genus *Quercus*. Despite extensive morphological research on *Cyclobalanopsis*, additional molecular data is essential to fully understand interspecific relationships and the broader phylogenetic framework within the genus, especially given the observed gene introgression and similarities in leaf characteristics among different sections (Li et al., 2024).

Chloroplasts, renowned for their semi-autonomous nature within the cell, play crucial roles in photosynthesis and synthesizing essential compounds. Studies have confirmed the presence of a distinct genetic system within these organelles (Bendich, 1987; Neuhaus and Emes, 2000). In flowering plants, the cp DNA’s structure, composition, and layout remain consistent across various plant families. Yet, within species, variations are observed that result in the alteration of genes and introns, including their acquisition or loss, and structural modifications such as expansions, contractions, and reversals at the inverted repeat regions. Analyzing cp DNA contrasts offers invaluable insights into plant phylogeny and evolutionary trajectories. Furthermore, the cp genome is now recognized as a superior genomic resource for elucidating evolutionary links and species-specific diversities over traditional taxonomic methodologies (Liu X. et al., 2019).

Since the release of the *Q. rubra* cp genome in 2014, there has been a notable rise in the sequencing of cp genomes across different oak species. This pattern showcases rapid advancements in next-generation sequencing technologies as well as a growing enthusiasm for using cp genomes to establish evolutionary relationships (Alexander and Woeste, 2014; Jiang et al., 2021; Wang et al., 2021; Yang et al., 2021; Li et al., 2024). The cp genomes of only 16 species within the *Cyclobalanopsis* section, a subgenus within *Quercus*, have been fully sequenced and analyzed. The limited availability of information hinders the widespread use of phylogenetic studies and molecular identification within the *Quercus* genus. Therefore, expanding the research on cp genome sequences is imperative to clarify taxonomic ambiguities within *Quercus*.

In this study, we newly published the cp genome sequences of seven *Quercus* section *Cyclobalanopsis* species, including *Q. argyrotricha*, *Q. augustinii*, *Q. bambusifolia*, *Q. bella*, *Q. edithiae*, *Q. jenseniana*, and *Q. poilanei*. Then we downloaded three already published data from NCBI. They are: *Q. litseoides*, *Q. obovatifolia*, and *Q. saravanensis*. Using these ten cp genomes, we performed (1) the structure and gene annotation, (2) comparative genomics analyses, (3) selection pressures, (4) phylogenetic analyses and divergence time estimation. Our study will provide valuable material for phylogenetic analyses of *Quercus* section *Cyclobalanopsis* (Chen X. et al., 2023).

## Materials and methods

### Plant material and DNA sequencing

Tender, unwounded leaf of seven *Quercus* section *Cyclobalanopsis* species (*Quercus argyrotricha*, *Q. augustinii*, *Q. bambusifolia*, *Q. bella*, *Q. edithiae*, *Q. jenseniana* and *Q. poilanei*) were harvested from four provinces in China: Yunnan, Hainan, Guangxi and Guizhou. Silica gel was used to dry the materials collected. Voucher specimens were saved at China West Normal University (CWNU), and sample information is listed in **Supplementary Table S1**. Associate Professor Xuemei Zhang of CWNU identified these specimens. Shanghai Tianhao Genomics Company carried out the extraction and sequencing of cp genomic DNA. The extracted DNA underwent double-ended sequencing through Illumina NovaSeq platforms. Quality control on the raw data used FastQC (Lo and Chain, 2014). For junction-dependent sequencing technologies (e.g., Illumina), special attention must be paid to removing contaminating junctions. Tainted junctions are located at the 3’end of the sequencing reads. So we use Adapter Removal to remove junction contamination at the 3’end; then we use the sliding window method for quality filtering.

### Cp genome assembly and genes annotation

The filtered readings were assembled using GetOrganelle v1.7.6.1 (Jin et al., 2020) to obtain the cp genome, with *Q. kerrii* (sequence number OP679796.1) as the reference sequence. The assembled cp genome was then annotated using GAVAS2 (http://47.96.249.172:16019/analyzer/annotate) (Shi et al., 2019). To ensure accuracy, the annotation process included manual corrections using Geneious R9.0.2 (Kearse et al., 2012) software, which incorporated start and stop codon positions and intron and exon boundaries. To enhance the precision of the annotation outcomes, a comparative study was conducted with closely related species. The annotated sequences of the cp genome were then uploaded to the U.S. National Center for Biotechnology Information (NCBI) database for analysis. The cp genome sequences map was drawn on OGDRAW (http://47.96.249.172:16085/CPGView/home) (Lohse et al., 2007).

### SSR and sporadic repeat sequences

The study examined scattered repetitive sequences in the cp genomes of ten different section of *Cyclobalanopsis* species using the online software REPuter (https://bibiserv.cebitec.uni-bielefeld.de/reputer/) (Kurtz et al., 2001). The analysis investigated forward (F), reverse (R), palindrome (P), and complementary (C) repeats. Various parameters were employed, including a minimum repeat length of 30 and a Hamming distance of 3, requiring repeat sequences to have a minimum similarity of 90%. The default settings were used for the other parameters, with 1000 parameters being configured. For the analyses Simple Sequence Repeats (SSRs), MISA (https://webblast.ipk-gatersleben.de/misa/) (Beier et al., 2017) software was utilized. Different cut-off points were used for diverse nucleotide repeats, encompassing parameters like 1-10 (single nucleotide repeats happening at least ten times), 2-5, 3-4, 4-3, 5-3, and 6-3 for SSR analysis. All the other parameters stayed in their default states. Manual verification was carried out on all the analyzed repetitions, and any redundant outcomes were removed (Li et al., 2024).

### Codon bias analysis

This research initially sifted through 52 distinctive non-repetitive sequences, each surpassing 300 base pairs and containing the ATG start codon, to get ready for subsequent analysis. We used the CodonW 1.4.2 program (Peden, 1997) to calculate various codon usage indices and base composition statistics for each coding sequence. The study involved analyzing various measurements related to the utilization of codons, such as relative synonymous codon usage (RSCU), codon adaptation index (CAI), optimal number of codons (ENC), index of codon bias (CBI), incidence of preferred codons (FOP), ENC scores, RSCU scores, and the likelihood of each nucleotide appearing in the third position of a codon (Li et al., 2024). The coding sequences’GC1, GC2, and GC3 contents were determined using EMBOSS software (Rice et al., 2000).

### Sequence variation of cp genome

To assess gene rearrangements and boundaries within the large single copy (LSC), small single copy (SSC), and inverted repeat (IR) regions of ten section *Cyclobalanopsis* species, the researchers utilized the IRscope (https://github.com/Limpfrog/irscope) (Amiryousefi et al., 2018) online tool to generate horizontal visualizations. They carried out a comparison analysis of sequence variations among ten genomes by employing the MVISTA software in shuffle-LAGAN mode. The MVISTA program can be accessed at the following URL: (http://genome.lbl.gov/vista/mvista/submit.shtml). They assessed nucleotide variation within cp genomes by screening for sites with high variability using DNAsp6 software (Rozas et al., 2017) based on the nucleotide diversity index (π).

### Selection pressure and phylogenetic analyses

KaKs_Calculator (https://sourceforge.net/projects/kakscalculator2/) (Wang et al., 2010) was adopted to calculate the rate of nonsynonymous mutation (Ka) and synonymous mutation (Ks) in protein-coding genes. The results of Ka/Ks could be used to assess the role of selection for each gene in CPGs of 10 *Quercus* species.

A phylogenetic tree of the genus *Quercus* was established using Bayesian (BI) analysis based on cp genomic data to understand the phylogenetic relationships of the genus *Quercus*. This tree included four sections of the genus *Quercus* endemic to China, encompassing 27 species (All 27 species in **Supplementary Table S2**), Trigonobalanus doichangensis as an outgroup. Apply all selected cp genomic sequences to align MAFFT v7.427 (Katoh and Standley, 2013). Later, MrBayes v3.2.7 (Ronquist et al., 2012) was utilized to carry out the BI tree analysis based on the following processes: infer the best-fit nucleotide substitution model (GTR+F+I+G4) by Modeltest (Posada and Crandall, 1998) and PAUP (Matthews and Rosenberger, 2008). A Markov chain Monte Carlo (MCMC) analysis was run for 6,000,000 generations, sampling the tree every 1,000 generations and ignoring the initial 0.25 as the burnin score.

### Divergence time estimate

The estimation of differentiation times for plants in Section *Cyclobalanopsis* was conducted using the BEAST v1.10.4 (Suchard et al., 2018) software package. Both Section *Cyclobalanopsis* and its closely related taxa have a rich fossil record for temporal calibration. Two crown node calibrations from Hipp et al.’s (Hipp et al., 2020) study on oak fossil calibration were used to calibrate differentiation times. Cp genome sequences were inputted using BEAUti v1.10.4, with species taxa set based on the node age calibration. Taxon differentiation time points were calibrated with a Normal prior distribution and a Yule tree prior with an uncorrelated relaxed lognormal molecular clock. The Markov-Chain Monte Carlo (MCMC) chain length was set to 200 million, run twice independently, with parameter sampling every 1000 generations. The xml files were then imported into BEAST v1.10.4. The tree and log files were combined using LOGCOMBINER, and convergence was assessed using Tracer v1.7.2 to detect effective sample sizes (ESSs) for all parameters. A 20% burn-in was set in TreeAnnotator v1.10.4 to generate the Maximum Clade Credibility tree (MCC). The tree file was displayed and edited in FigTree v1.4, and the 95% confidence intervals (95% HPD) were examined.

## Result

### Basic properties of the cp genome

**Figure 1:**
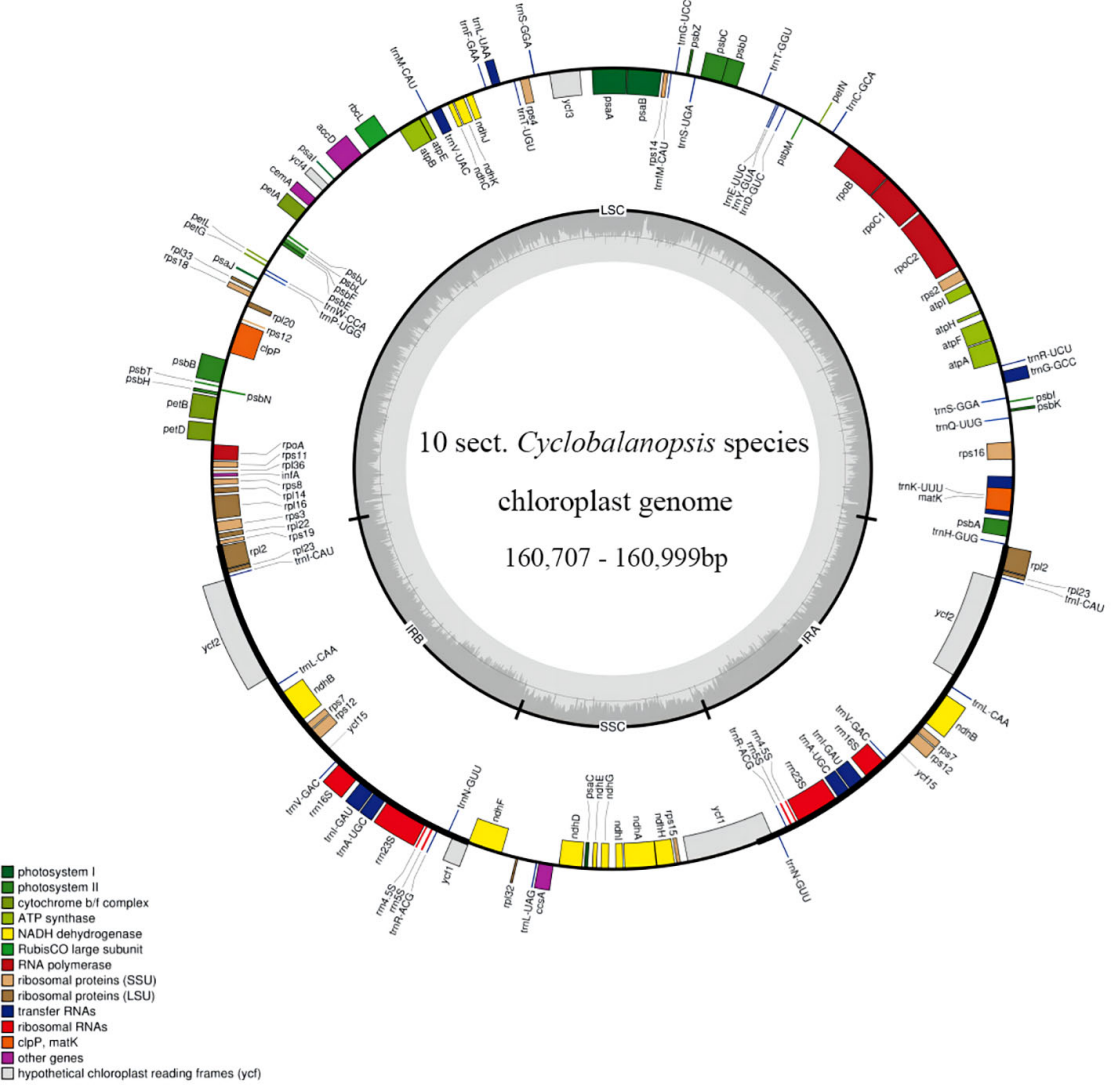
Gene map of ten Quercus section Cyclobalanopsis species. Genes within and outside the circle are transcribed in the clockwise and counterclockwise directions respectively. The darker and lighter grey in the inner circle respectively represent GC and AT content.

We managed to sequence the entire cp genomes of seven *Cyclobalanopsis* species (*Q. argyrotricha*, *Q. augustinii*, *Q. bambusifolia*, *Q. bella*, *Q. edithiae*, *Q. jenseniana*, and *Q. poilanei*). We compared them with the three published cp genomes sequences (*Q. litseoides*, *Q. obovatifolia*, and *Q. saravanensis*), whose lengths ranged from 160,707 to 160,999 base pairs (**Figure 1**). The genes were conserved in the composition and sequence within these cp genomes. We also found that these genomes have a very typical tetragonal structure, with an LSC region (90,147 bp to 90,326 bp) flanked by two IR regions (25,809 bp to 25,860 bp), separating it from the SSC region (18,788 bp to 18,993 bp). In all, 132 genes were identified, including 8 rRNA genes, 37 tRNA genes, and 87 protein-coding genes (**Table 1**).

**Table 1:** A summary of the statistics regarding the cp genomes of 10 *Quercus* sect. *Cyclobalanopsis* species.

Species	*Q. argyrotricha*	*Q. augustinii*	*Q. bambusifolia*	*Q. bella*	*Q. edithiae*	*Q. jenseniana*	*Q. litseoides*	*Q. obovatifolia*	*Q. poilanei*	*Q. saravanensis*
Genome size (bp)	160,999	160,846	160,803	160,798	160,707	160,802	160,782	160,817	160,784	160,767
Length of LSC (bp)	90,326	90,228	90,274	90,255	90,147	90,236	90,235	90,210	90,216	90,220
Length of IRs (bp)	25,840	25,860	25,811	25,833	25,842	25,842	25,840	25,809	25,842	25,825
Length of SSC (bp)	18,993	18,898	18,907	18,788	18,876	18,882	18,867	18,989	18,884	18,897
Number of genes	132	132	132	132	132	132	132	132	132	132
protein-coding genes	87	87	87	87	87	87	87	87	87	87
tRNA genes	37	37	37	37	37	37	37	37	37	37
rRNA genes	8	8	8	8	8	8	8	8	8	8
GC content (%)	36.90	36.89	36.88	36.89	36.90	36.89	36.90	36.90	36.89	36.89
GC content of LSC (%)	34.78	34.75	34.73	34.74	34.75	34.74	34.74	34.75	34.74	34.74
GC content of IRs (%)	42.77	42.75	42.79	42.77	42.77	42.77	42.77	42.78	42.77	42.76
GC content of SSC (%)	30.97	31.09	31.06	31.11	31.12	31.10	31.13	31.10	31.11	31.08
Species origin	new sequencing	new sequencing	new sequencing	new sequencing	new sequencing	new sequencing	NC_065782	NC_039972	new sequencing	NC_060377

### Codon usage

**Figure 2:**
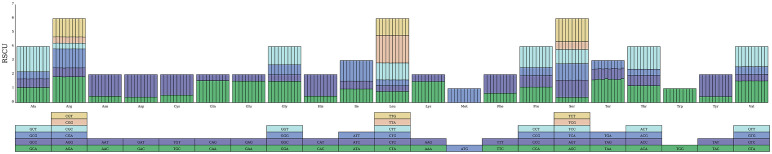
The RSCU of amino acids in 10 section Cyclobalanopsis cp genomes. Boxes beneath the graphs represent all the codons encoding each amino acid. The colors of the histograms correspond to those of the codons.

The codon usage patterns were presented in **Table 2**. The protein-coding genes, spanning from 62,493 to 62,991 bp, were obtained from ten species in the section *Cyclobalanopsis*. The quantities of synonymous codons fluctuated from 20,831 to 20,992. The ENC (Effective Number of Codons) value lay between 49.89 and 49.96. The FOP (Frequency of Optimal Codons) value was 0.355 in *Q. augustinii*, *Q. bella* and 0.354 in the other eight samples. The GC content was between 37.94 and 38.00%. The codon preference indexes of the ten species varied slightly, indicating similar codon usage. The GC3 of ten species ranged between 29.85 and 29.94%. The analysis of codon usage preference in the *Cyclobalanopsis* cp genomes revealed 30 high-frequency codons with Relative Synonymous Codon Usage (RSCU) values greater than 1, of which 28 ended with A or U bases and only two with G or C bases. This demonstrates a bias towards A/U endings in the *Cyclobalanopsis* cp genomes. Nonetheless, the tryptophan codon (UGG) and the methionine codon (AUG) did not exhibit a clear preference, as indicated by their RSCU values. **Figure 2** shows a significant conservation of codon usage within the section *Cyclobalanopsis*, although species-specific variations were present (Specific RSCU values are shown in **Supplementary Table S3**).

**Table 2:** Codon preference indicators of 10 species of section *Cyclobalanopsis*.

Index	Q. argyrotricha	Q. augustinii	Q. bambusifolia	Q. bella	Q. edithiae	Q. jenseniana	Q. litseoides	Q. obovatifolia	Q. poilanei	Q. saravanensis
Length (bp)	62,991	62,931	62,829	62,976	62,838	62,940	62,838	62,493	62,940	62,823
Codon number	20,980	20,977	20,943	20,992	20,946	20,980	20,946	20,831	20,980	20,941
Effective number of codons	49.89	49.94	49.94	49.94	49.96	49.92	49.95	49.95	49.91	49.94
Codon adaptation index	0.167	0.167	0.167	0.167	0.167	0.167	0.167	0.167	0.167	0.167
Codon bias index	-0.099	-0.099	-0.099	-0.099	-0.100	-0.099	-0.100	-0.100	-0.100	-0.099
Frequency of optimal codons	0.354	0.355	0.354	0.355	0.354	0.354	0.354	0.354	0.354	0.354
GC content (%)	37.94	37.96	37.97	37.96	37.99	37.95	37.98	38.00	37.95	37.97
GC1 content (%)	46.12	46.14	46.16	46.11	46.18	46.13	46.19	46.24	46.12	46.17
GC2 content (%)	37.86	37.85	37.83	37.85	37.84	37.85	37.83	37.85	37.85	37.83
GC3 content (%)	29.85	29.89	29.92	29.91	29.94	29.88	29.92	29.92	29.89	29.91

### Analysis of the cp genome structure

**Figure 3:**
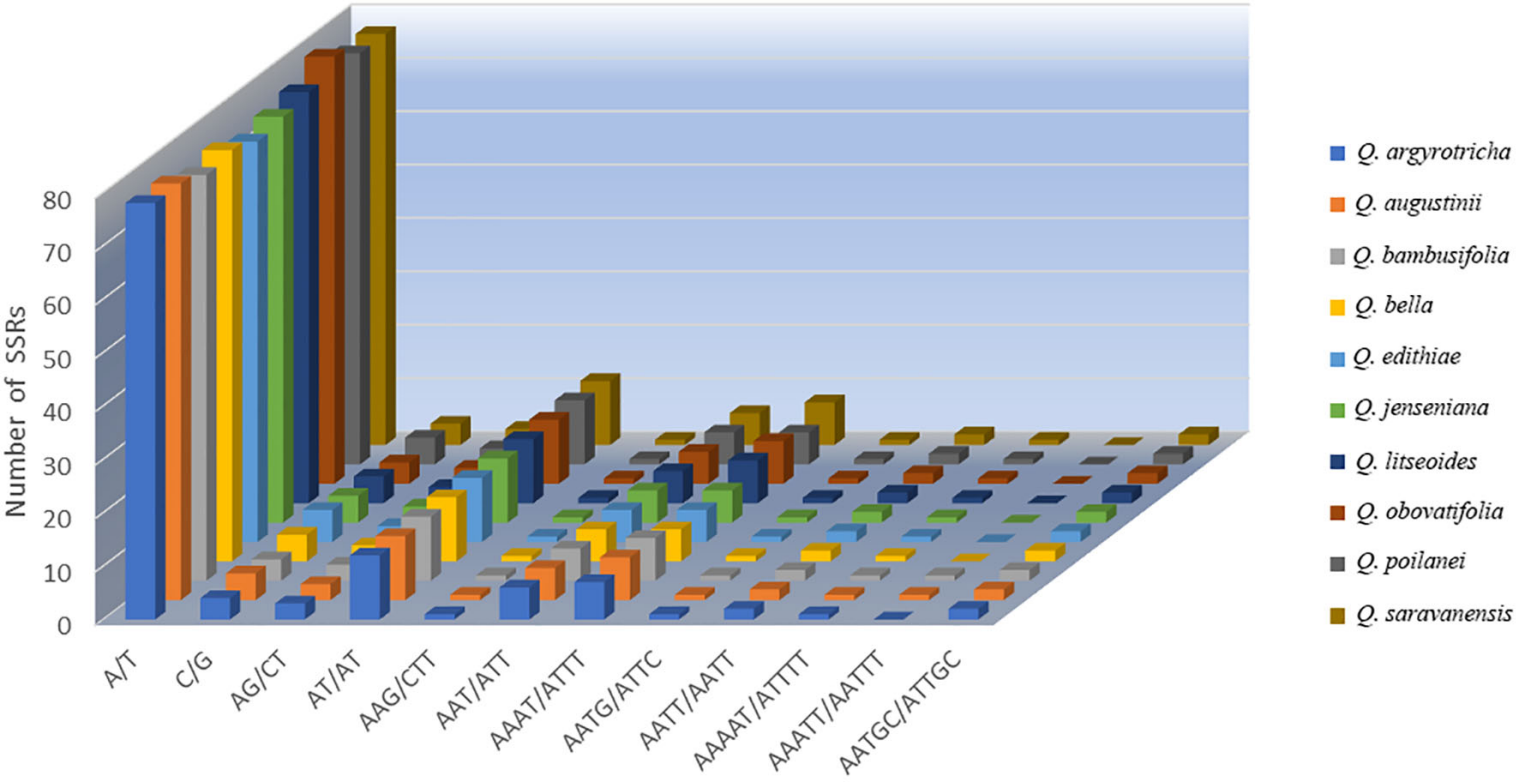
The number of SSR units detected in 10 section Cyclobalanopsis cp genome.

**Figure 4:**
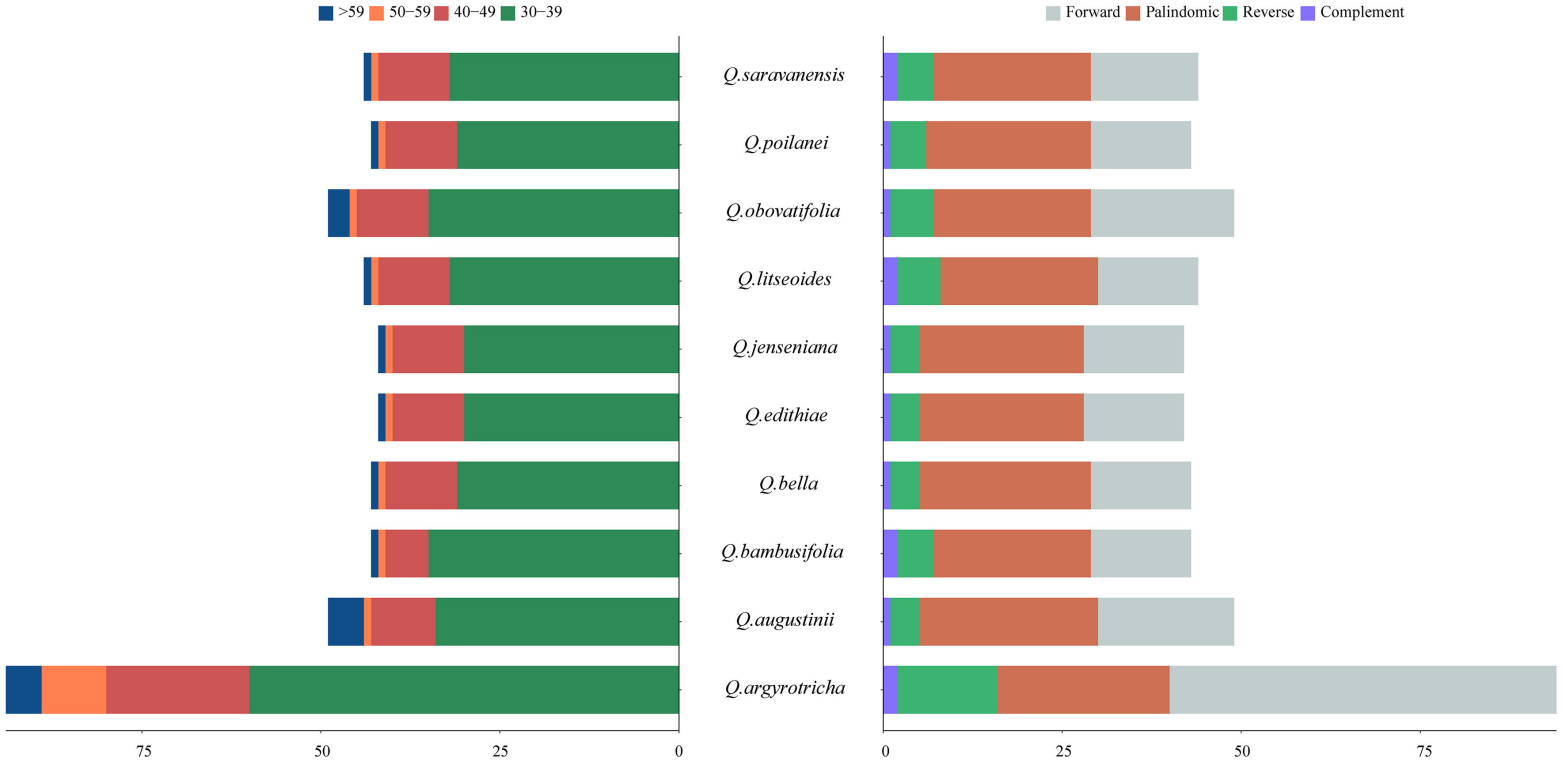
Type and quantity of scattered repeats in the cp genome of 10 section Cyclobalanopsis cp genomes.

The SSR analysis detected five types of SSRs: single, dinucleotide, trinucleotide, tetranucleotide, and pentanucleotide repeats. The cp genomes of the ten plant species mainly had mononucleotide SSRs, and pentanucleotide SSRs were the least common. *Q. augustinii* and *Q. obovatifolia* had the greatest number of SSRs (120), while *Q. edithiae* and *Q. jenseniana* had the lowest (115). The difference in the number of SSRs among the species was not statistically significant. Among the ten *Cyclobalanopsis* species, A/T simple repeats were the most frequent, with AAG/CTT, AATG/ATTC, and AAAAT/ATTTT repeats each occurring only once. Uniquely, the SSR AAATT/AATTT was only identified in *Q. augustinii* and *Q. bambusifolia* (**Figure 3** and **Supplementary Table S4**). The search for dispersed repetitive sequences uncovered four kinds: palindromic (P), complement (C), reverse (R), and forward (F). Although there were minor variations in their quantities, P were the most abundant, while C were the least known (**Figure 4** and **Supplementary Table S5**). Notably, *Q. argyrotricha* had more repetitive sequences.

**Figure 5:**
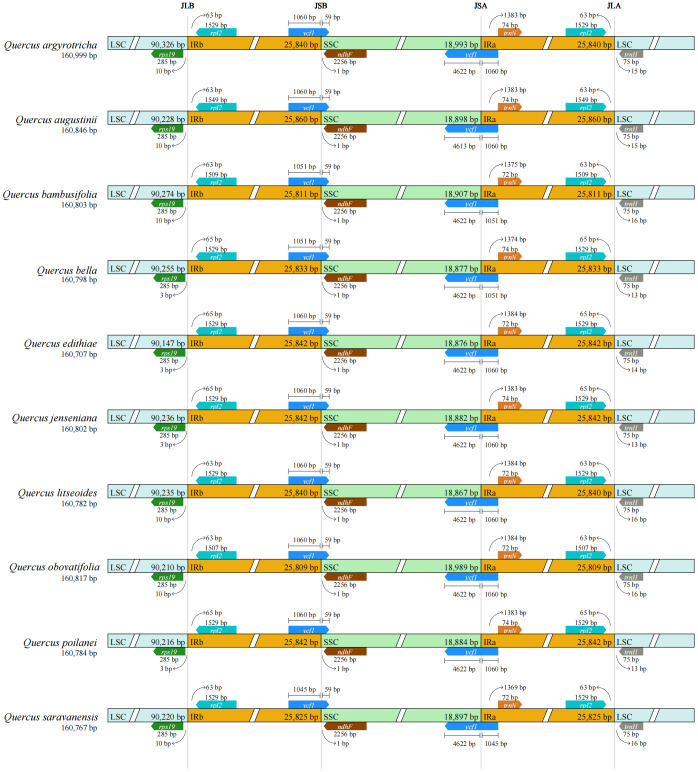
LSC, SSC and IR boundaries of the cp genomes in 10 section Cyclobalanopsis cp genome.

A common feature found in the cp genomes of the ten species belonging to section *Cyclobalanopsis* was the presence of a circular tetrad structure defined by four distinct boundaries: IRa-LSC, SSC-IRa, IRb-SSC, and LSC-IRb (**Figure 5**). While the genome sizes of these species were similar, with minor variations, there were differences observed in the genes flanking these regions, specifically *rps19*, *ndhF*, and *ycf1*. It was noted that *rps19* consistently resided within the LSC region, albeit at varying distances from the LSC-IRb boundary, specifically at 3 bp or 10 bp. The genes *ndhF* and *ycf1* were positioned in close proximity to the SSC-IR boundaries. Furthermore, a segment of *ycf1* of the same length was detected in the IRb region of all ten *Cyclobalanopsis* species, however, it was categorized as a pseudogene (*ψycf1*).

### Sequence divergence, hotspots and selection pressure estimation

**Figure 6:**
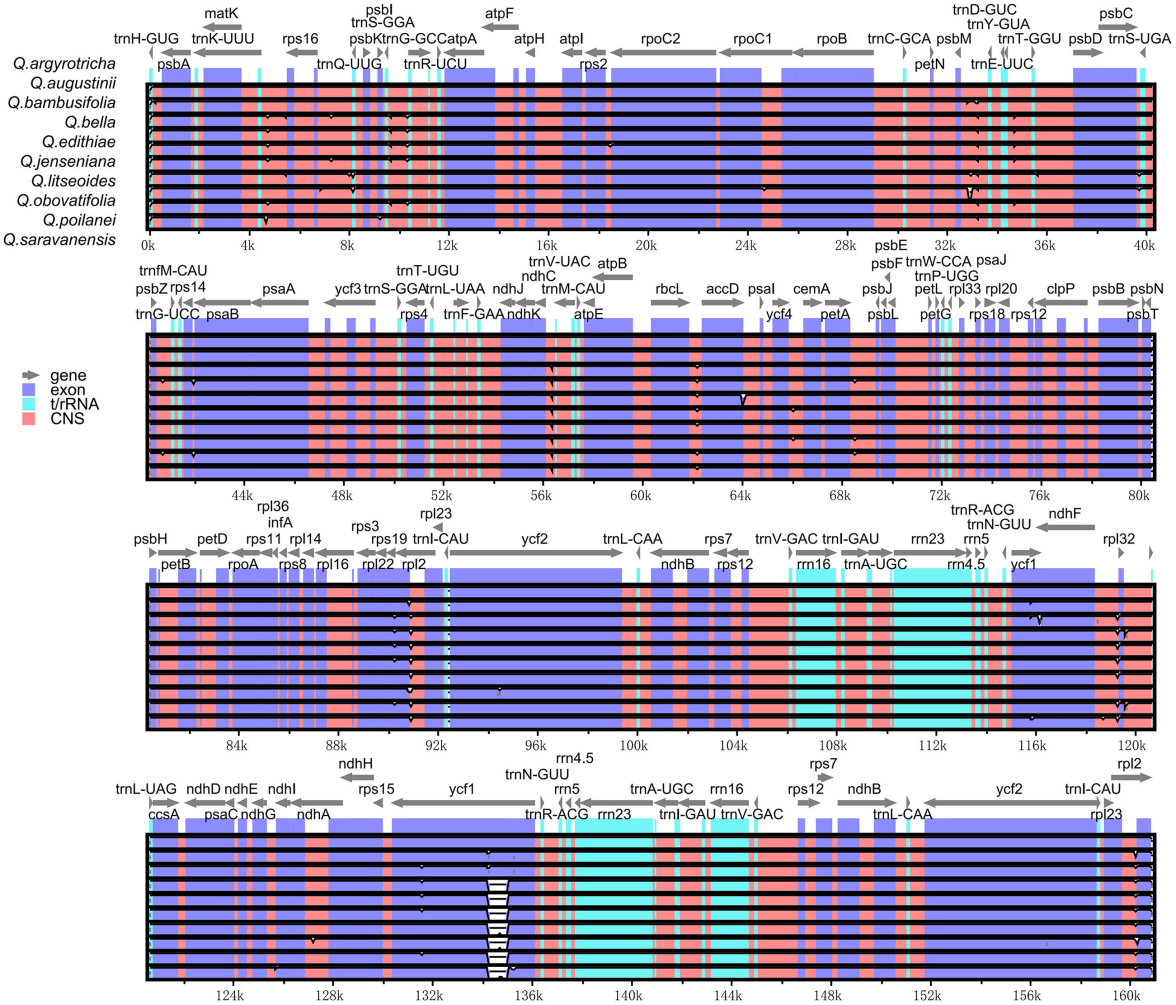
Comparison of cp genomes among 10 section Cyclobalanopsis species.

**Figure 7:**
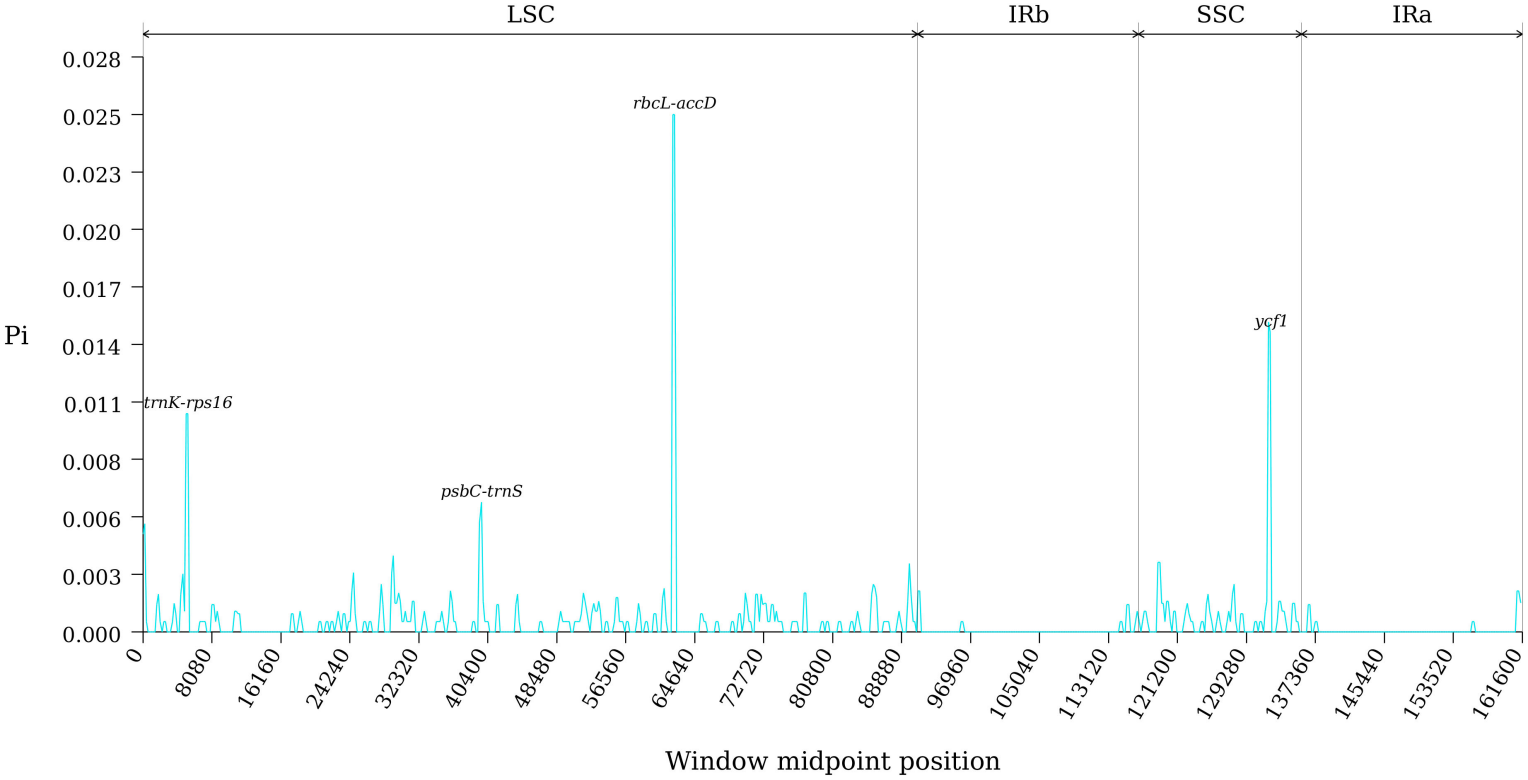
Comparison of nucleotide variability of common genes in 10 species of section Cyclobalanopsis.

The study conducted an analysis of sequence variation in the cp genomes of ten species within section *Cyclobalanopsis* using the Shuffle-LAGAN model in the mVISTA online software, with the cp genome sequence of *Q. kerrii* as a reference. Results depicted in **Figure 6** revealed high conservation in the coding regions of the cp genomes of the ten *Cyclobalanopsis* species, with minimal variation in the rRNA genes. Despite the typical conservation of the cp genome in section *Cyclobalanopsis*, some differences were observed across various groups. Nucleotide polymorphisms were identified in the cp genes of the ten *Cyclobalanopsis* species. **Figure 7** displayed a relatively higher sequence conservation in the intergenic spacer of the reverse repeat (IR) region compared to the single-copy region. Pi values can be found in **Supplementary Table S6**. The study then identified the *trnK-rps16*, *psbC-trnS*, *rbcL-accD*, and *ycf1* genes (pi > 0.006) from the coding regions as potential DNA barcodes for investigating the genetic and phylogenetic relationships among *Quercus* species.

**Figure 8:**
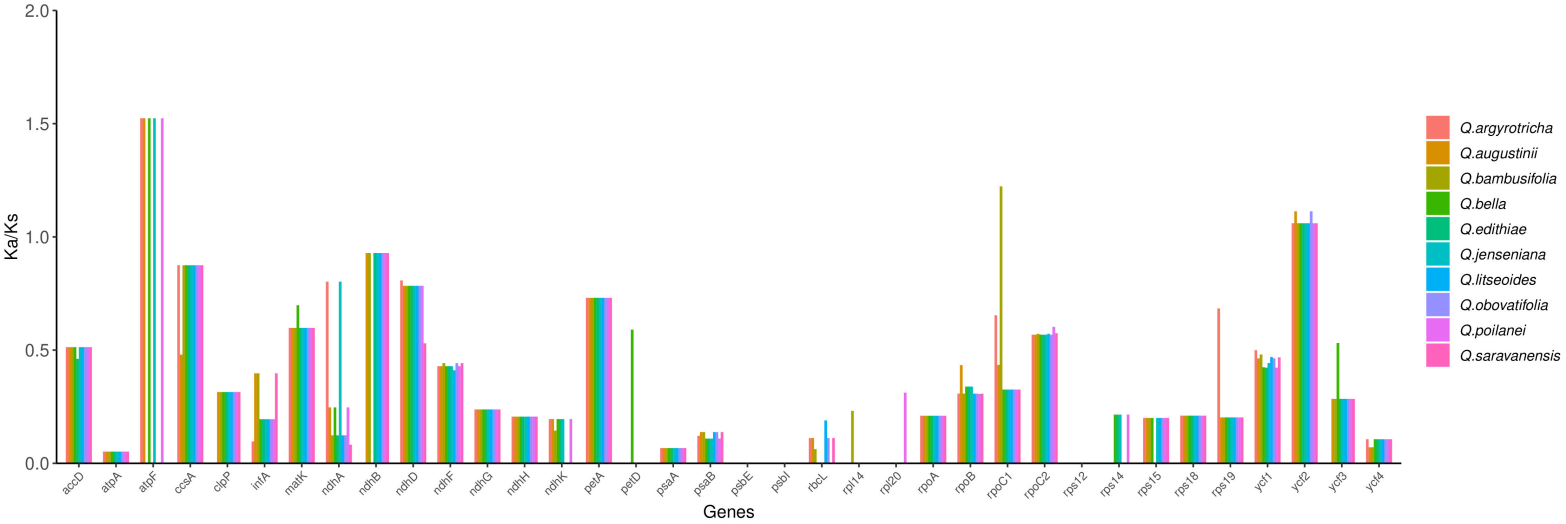
Ka/Ks of 36 protein-coding genes in 10 section Cyclobalanopsis cp genome.

To estimate the role of selection of the *Quercus* section *Cyclobalanopsis* species, Ka and Ks values in 10 cp genomes using *Quercus chenii* as a reference. The Ka/Ks values were calculated and recorded in **Supplementary Table S7**, ranging from 0 to 1.52384. Among these, 36 protein-coding genes showed significance (**Figure 8**) in 10 species. Based on the calculation results, we speculated that the purification selection may have affected most protein-coding genes, as their Ka/Ks values were less than 1. At the same time, when Ka/Ks > 1 demonstrated that the positive selection was working on the genes. Therefore we identified three genes that were under the positive selection, namely the *atpF* gene in *Q. argyrotricha*, *Q. augustinii*, *Q. bella*, *Q. jenseniana*, and *Q. poilanei*, *rpoC1* gene in *Q. bambusifolia* and *ycf2* gene in all 10 species.

### Phylogenetic relationships

**Figure 9:**
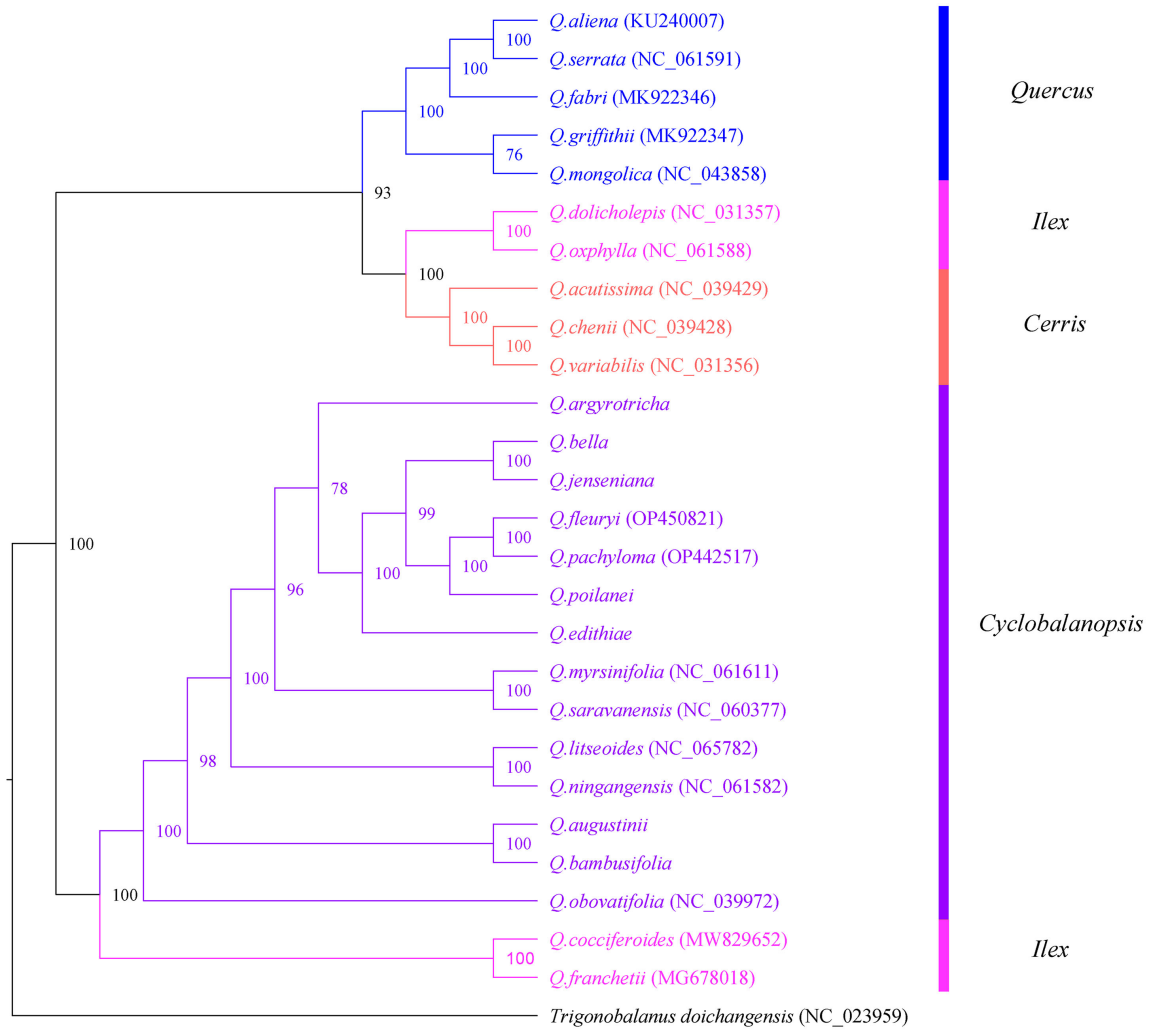
Bayesian(BI) analysis phylogenetic tree among 27 cp genomes of Fagaceae species. Values above the branch represented bootstrap support.

To explore the phylogenetic relationships among species of Section *Cyclobalanopsis*, 7 species assembled and annotated in this study were combined with 20 species downloaded from NCBI to construct a phylogenetic tree. This study used Bayesian inference (BI) to build a phylogenetic tree (**Figure 9**). Based on the phylogenetic tree, *Trigonobalanus doichangensis* was located at the base of the phylogenetic tree and represents an early divergent group within the Fagaceae family. The phylogenetic tree consists of two main branches: Subgenus *Cerris* and Subgenus *Quercus*. The former category includes 14 different species belonging to Section *Cyclobalanopsis*, 4 from Section *Ilex*, and 3 from Section *Cerris*. The latter shall consist of 5 species from Section *Quercus*. All branches had support rates ranging from moderate (greater than 70%) to advanced (greater than 90%), and most nodes had support rates of 100% (All 27 species in **Supplementary Table S2**).

### Divergence time estimate

**Figure 10:**
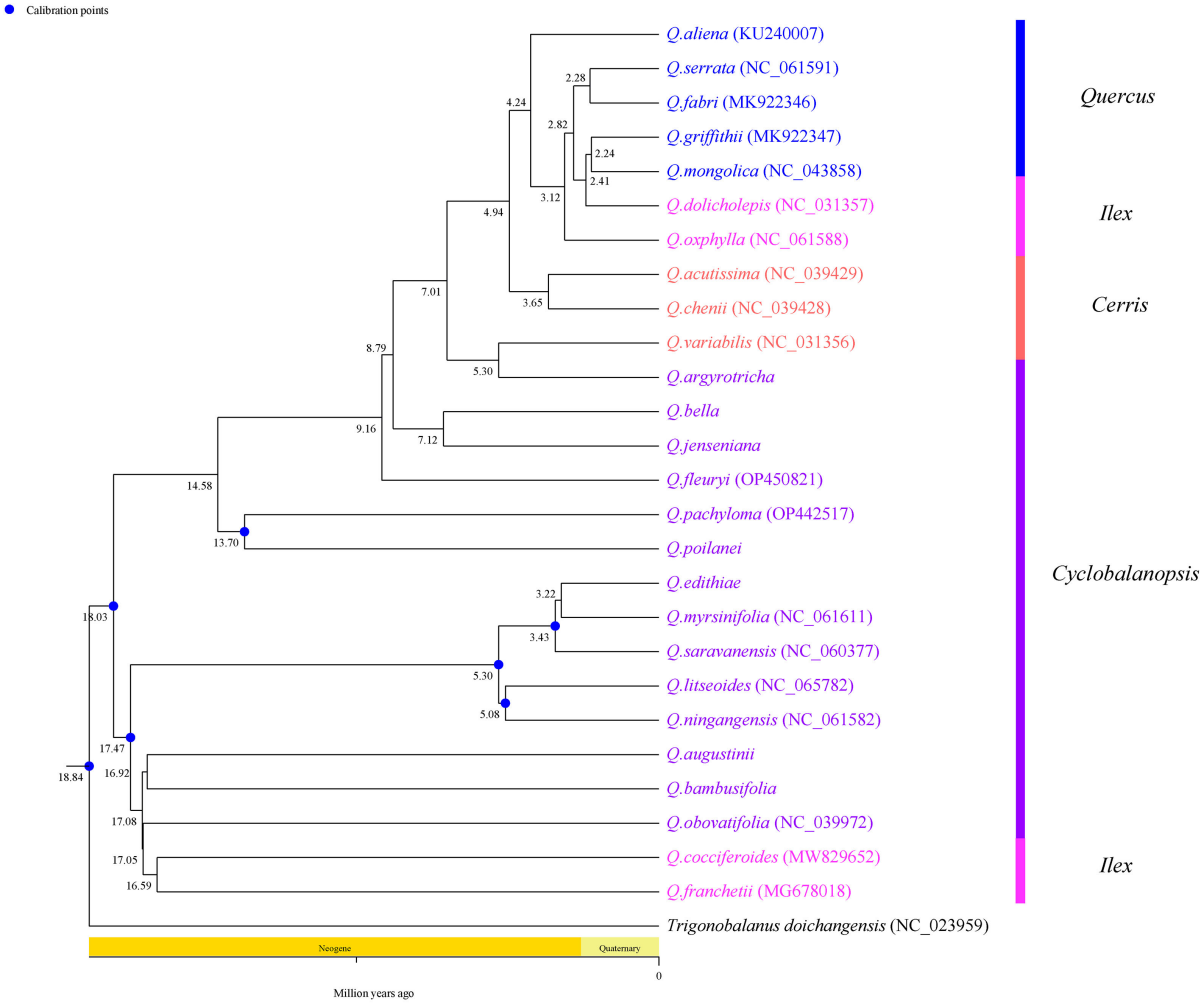
Analysis of divergence times based on chloroplast genomes in Sect. Cyclobalanopsis. The value on the node is node ages (million years ago, Ma).

This study used phylogenetic species to estimate the differentiation time of Section *Cyclobalanopsis* species based on chloroplast genome sequences (**Figure 10**). The results showed that the most recent common ancestor of 14 Section *Cyclobalanopsis* plants can be traced back to Neogene (18.03 Ma), which was earlier than the divergence time of other taxa. This study demonstrated that Section *Cyclobalanopsis* is the earliest occurring type in the genus *Quercus*.

## Discussion

### Cp genome architectures in ten *Quercus* section *Cyclobalanopsis* species

We successfully constructed the cp genomes of seven *Quercus* section *Cyclobalanopsis* species and downloaded three species from NCBI for joint analysis. The size of the 10 cp genomes (ca. 160 kb) is consistent with the plastid chromosomes of photosynthetic land plants, which range in size from 120 to 160kb (Wicke et al., 2011). The same quadripartite circular structure was found in the ten *Quercus* section *Cyclobalanopsis* species (Li et al., 2018; Wang et al., 2021; Chen et al., 2022). Moreover, the GC content is an essential indicator of phylogenetic relationships between species (Mitreva et al., 2006). Therefore, the overall GC content in the IR and SC regions of ten *Quercus* species was analyzed. The results suggest that the overall GC content and the GC content within the IR and SC regions are largely consistent, with the GC content in the intergenic spacer (IGS) region showing a statistically significant rise compared to the GC content in the large single-copy (LSC) region and the small single-copy (SSC) region (Li et al., 2024).

The position of boundaries between cp sequences is a critical component in the study of cp genome evolution, and the contraction and expansion of IR boundaries can help elucidate phylogenetic relationships between different taxa (Chen J. et al., 2023; Huang et al., 2022). Based on common evolutionary phenomena in plant genomes, we must be aware that the position of the IR/SC boundary may change due to contraction or expansion of the IR region (Wang and Messing, 2011; Huang et al., 2014). The IRs/SCs boundaries of all species compared in this study are located in similar positions, with slight differences in displacement between species. The conservatism of the *Quercus* section *Cyclobalanopsis* was demonstrated by the relatively constant length of cp genomes and the minor variations in their region borders, which are the same conditions as other *Quercus* species (Xu et al., 2015; Wang et al., 2021).

The preference for codon usage is a key aspect of biological evolution, shaped by a variety of factors that influence genetic code functionality. These influencing factors include genome size, base mutations, genetic drift, natural selection, gene expression levels, and protein structure (Angellotti et al., 2007). It is important to recognize that synonymous codons arise from mutations, and their relative usage can be assessed using the measure of relative synonymous codon usage (RSCU). The RSCU provides insights into differences in codon preference across various genes (Parvathy et al., 2022). By analyzing RSCU, we observed 64 codons with a distinct preference for A/T-ending sequences in these oak species. Interestingly, this pattern was also evident in an analysis of GC3 content, indicating a predilection for A/T-ending codons in the *Quercus* genus studied. This phenomenon of codon usage bias is frequently observed in angiosperms (Li et al., 2024).

### Diversity of repetitive sequences and SSRs

Repetitive sequences play a crucial role in storing genetic information, influencing gene expression, and impacting the inheritance and evolution of plant species (Moore et al., 2010). Our study examined ten species within the *Cyclobalanopsis* section and found that the number of simple sequence repeats (SSRs) ranged from 115 to 120. Single nucleotide repeats were the most common type of SSRs, followed by dinucleotide and tetranucleotide repeats. The SSRs in *Cyclobalanopsis* cp genomes had a high A/T base content, indicating a preference for A/T bases. Interestingly, no hexanucleotide repeats were found, consistent with previous research on *Cyclobalanopsis* section (Wang et al., 2021; Li et al., 2022). Dispersed repeats were also observed in the ten distinct species of *Cyclobalanopsis* section, consisting mainly of forward and palindromic sequences. However, differences in the number of tandem repeats among the species suggested varying rates of mutational events (Li et al., 2024).

### Conservatisms, highly variable regions and selection pressure estimation

Our study compared 10 species by mVISTA sequence identity analysis using the *Q. kerrii* genome as a reference. The results showed that these cp genomes exhibit high conservation in coding regions and significant differences in non-coding regions, especially in the LSC and SSC regions. This is similar to the findings of most angiosperm cp genomes showing high regional diversity (Xu et al., 2020; Fan et al., 2021, 2022). The non-coding regions showed a high degree of variation, especially in the LSC and SSC regions. *Q. bella*, *Q. edithiae*, *Q. jenseniana*, *Q. litseoides*, *Q. obovatifolia*, *Q. poilanei* and *Q. saravanensis* showed significant sequence variation in the *ycf1* gene region. Since the role of the *ycf1* gene in cp function is not fully understood, these variations may reflect the evolution of specific adaptations in these species. Sliding window analysis by dnaSP v6 software revealed the nucleotide diversity values (Pi) range in the cp genomes of 10 plant species. Four highly mutated regions were screened, namely *trnK-rps16*, *psbC-trnS*, *rbcL-accD* and *ycf1* genes. These highly variable regions provide a valuable resource for developing molecular markers. Because of their genetic diversity, they are ideal candidates for developing species-specific DNA barcodes (Kress, 2017; Antil et al., 2023).

In our study, most of the Ka/Ks values were less than 1 or unavailable, suggesting that the emergence frequency of synonymous nucleotide substitution was more than that of non-synonymous nucleotide substitution due to the purify selection process (Matsuoka et al., 2002; Castle, 2011). Therefore we identified three genes that were under the positive selection, namely the atpF gene in *Q. argyrotricha*, *Q. augustinii*, *Q. bella*, *Q. jenseniana*, and *Q. poilanei*, *rpoC1* gene in *Q. bambusifolia* and *ycf2* gene in all 10 species. Whether these divergence hotspots found in the above analysis could be utilized for DNA barcodes or estimating taxonomic evolution in genus *Quercus* needs further research.

### Inference of phylogenetic relationship

China is one of the centers of diversity of the subgenus *Cyclobalanopsi* and presents significant challenges in understanding the evolution of oak species (Carrero et al., 2020). Studies of the taxonomy based on the morphology of oak trees are limited due to convergent evolution and the common occurrence of hybridization among different species. In spite of these challenges, Deng Min successfully developed a classification system for the *Cyclobalanopsis* section. However, the only molecular phylogenetic analysis of the genus *Quercus* in China has depended on RAD-seq sequencing (Deng et al., 2018). Most studies utilizing cp genomes have successfully yielded high-resolution and well-supported phylogenetic trees, even in phylogenetically challenging plant taxa (Feng et al., 2022; Lee et al., 2022; Feng et al., 2023; Li et al., 2023). The phylogenetic tree comprises two primary branches: Subgenus *Cerris* and Subgenus *Quercus*. The first group encompasses fourteen distinct species within Section *Cyclobalanopsis*, four species from Section *Ilex*, and another three species from Section *Cerris*. The latter shall consist of five species from Section *Quercus*—the species of Sect. *Quercus* forms a separate branch, whereas the species of Sect. *Cyclobalanopsis*, Sect. *Ilex* and Sect. *Cerris* forms paraphyletic groups. It has been found in existing studies of the genus *Quercus* that the reason Sect. *Ilex* is not a monophyletic group, possibly due to incomplete genealogical screening or gradual infiltration between ancestral taxa (Hipp et al., 2020).

Further investigation of species within the *Cyclobalanopsis* section revealed that *Q. bella* and *Q. jenseniana* formed sister branches, while *Q. augustinii* and *Q. bambusifolia* also clustered together. However, a previous study by Deng Min et al. (2014) they were proposed that *Q. jenseniana* might be more closely related to *Q. augustinii*. This discrepancy could be due to two main reasons: first, Deng Min et al. primarily classified the species based on morphological characteristics, such as leaf epidermis, where *Q. jenseniana* and *Q. augustinii* share similarities. Second, there might have been genetic introgression, as *Q. jenseniana* was collected from the same location as *Q. bella*. Collecting samples of *Q. jenseniana* from various populations and geographic ranges is recommended for further analysis of their morphological traits. Interestingly, *Q. argyrotricha* does not group with the other species in the phylogenetic tree, contradicting previous studies based on morphology and molecular phylogenetics. We propose that this discrepancy in the relationship and systematic position of *Q. argyrotricha* could be attributed to unique repetitive sequences in its cp genome compared to other species, as well as its higher altitude of collection. *Q. argyrotricha* displays the highest number of repetitive sequences among the analyzed species, suggesting potential recombination events and greater genetic diversity.

Trichomes are typically located on the leaves, particularly during the early stages of leaf development. Some trichomes shed and vanish as the leaf matures, leaving only their bases on the leaf surface. This characteristic holds significant taxonomic importance in the Quercus genus. Plants in the Cyclobalanopsis section generally possess unicellular and composite trichomes, with the former being more prevalent. In this study, all species except Q. argyrotricha exhibited unicellular trichomes, aligning with previous findings (Deng et al., 2014). Although genomic data could potentially aid in resolving species classification challenges within Quercus, it is noted that the chloroplast genome represents only a portion of the plant’s total genetic material, underscoring the issue’s complexity. Therefore, developing advanced genetic techniques for phylogenetic and population analysis is expected to deepen our insights into the evolutionary history of the Quercus genus.

### Divergence time estimate

Thanks to the matrilineal nature of inheritance, cp genomic data are relatively stable, and their distribution is not prone to change in any species, allowing them to be effectively utilized to study the evolutionary history of species (Daniell et al., 2016). In this study, we estimated species divergence time based on the chloroplast genome and showed that the Section *Cyclobalanopsis* branch can be traced back to Neogene (18.03 Ma). Through the study of the world’s first fossil of Section *Cyclobalanopsis* from the Maoming Basin, Guangdong Province, China, the previous authors concluded that Section *Cyclobalanopsis* plants reached the tropical and subtropical regions of South China in the Early Oligocene, which also suggests that the modern distribution pattern of Section *Cyclobalanopsis* may have originated in the Oligocene (Liu X. Y. et al., 2019). Fossil evidence indicates that the genus Palaeocrustacea is the earliest-appearing taxon of the genus *Quercus*, and its leaf structure and cuticle characteristics are closest to those of Section *Cyclobalanopsis*, so it can be concluded that Section *Cyclobalanopsis* is the earliest-appearing type of the genus *Quercus*. In the present study, this argument can also be proved. In this study, Section *Cyclobalanopsis* mainly diverged in Neogene (ca. 3.22-18.03 Ma), a period of rapid uplift of the Tibetan Plateau (17-25 Ma) (Wen et al., 2014; Favre et al., 2015), which may have allowed species from Eurasia to diverge in response to geographic and environmental isolation (Li et al., 2019). In addition, the formation of the East Asian summer monsoon system was an essential climatic event during this period, which brought abundant precipitation and facilitated the lineage differentiation of plant groups (Sun and Wang, 2005; Kong et al., 2017). In this study, Section *Cyclobalanopsis* plants successively diverged in 17-25 Ma, reflecting the diversification of Section *Cyclobalanopsis* species, which is close to previous estimates based on the RAD-seq dataset (ca 10 Ma) (Hipp et al., 2020). The rapid species differentiation during this time was mainly driven by Himalayan movements and climate change, such as the rapid uplift of the Tibetan Plateau around 13-15 Ma, 7-8 Ma, and 1.6-3.5 Ma, and the significant increase in the strength of the East Asian monsoon during the same period, which drove the rapid differentiation of plant taxa (Sun and Wang, 2005; Wen et al., 2014).

## Conclusion

In this study, we sequenced seven species in *Quercus* section *Cyclobalanopsis* and downloaded three species from NCBI for analysis together. Cp genome assembly and annotation revealed that the cp genome of *Quercus* plants has a typical circular tetrameric structure, and the cp genome size ranges from 160,707 to 160,999 bp. By comparative genome analysis, the species we studied prefer codons ending in A/U. The most numerous simple repeat sequences in *Quercus* plants are single nucleotides, mainly A/T-based. There was no obvious contraction and expansion of the IR/SC boundary in these plants. Only *ycf1* gene showed *ψycf1* gene in the IRB/SC boundary. Four highly variable hotspots were detected in comparison analysis, they are *trnK-rps16*, *psbC-trnS*, *rbcL-accD*, and *ycf1*. Besides, three genes (*atpF*, *rpoC1*, and *ycf2*) were detected under positive selection pressure. Phylogenetic analysis shows that *Q. bella* and *Q. jenseniana*, *Q augustinii*, and *Q. bambusifolia* have a recent relationship. It is noteworthy that *Q. argyrotricha* is individually differentiated, possibly related to its repetitive sequence differences. Divergence time analysis revealed that Section *Cyclobalanopsis* represents the earliest type of *Quercus* genus. The findings obtained will facilitate further investigations into the taxonomy, phylogenetic evolution, and preservation of *Quercus* genus.

## Data Availability

The datasets generated and/or analyzed during the current study are available in the National Center for Biotechnology Information repository, Accession Number: OQ534364, PP498793, PP498794, PP471977, PP471975, PP498796, PP498797.
